# Substance use, anxiety and depression among Tunisian college students

**DOI:** 10.1192/j.eurpsy.2024.988

**Published:** 2024-08-27

**Authors:** E. Bergaoui, A. Bouallagui, M. Moalla, M. Zrelli, G. Amri, R. Ghachem

**Affiliations:** Psychiatry B, Razi hospital, Manouba, Tunisia

## Abstract

**Introduction:**

Substance use and mental health symptoms are frequent among college students worldwide.

**Objectives:**

This study examined the prevalence of substance use, anxiety and depression among college students and their associated factors.

**Methods:**

A total of 115 college students aged between 19 and 30 years from different universities completed a self-reported online survey during March 2023. The questionnaire included items on demographic information, substance use and the Hospital Anxiety and Depression scale.

**Results:**

Women represented 70% of our population. The average age was 25,1±3,5 years.

Twenty-nine (25,2%) were smokers, thirty-three students (28,7%) consumed alcohol and nine students (7,8%) used cannabis. Five students used ecstasy. Four students used LSD. Three students used cocaine.

Average HADS anxiety score was 7,96±4,26. Twenty-one students (18,3%) had mild anxiety symptoms. Thirty-three students (28,7%) had moderate to severe anxiety symptoms.

Average HADS depression score was 8,59±4. Thirty-one students (27%) had mild depressive symptoms. Forty-one (35,7%) had moderate to severe symptoms of depression.

Smoking was unrelated to gender, age, field of studies, economic or social status, family or personal history. It was related to drinking and doing other activities or hanging out with friends (p<0,001). Drinking alcohol was related to the field and year of study, age, hanging out with friends and other substance use (p<0,001). Using cannabis was related to psychiatric family history (p<0,05). Using one substance was related to using other substances (p<0,05). Anxiety was related to gender (p<0,001). Depression levels were related to socio-economic statuts (p=0,041). Poly-use was more frequent among older students (p=0,003) and medical students (p=0,031). Substance use was unrelated to anxiety and depression levels.

**Conclusions:**

Tunisian universisties should consider detecting students with substance and mental health problems and offer them support and treatment if needed.

**Disclosure of Interest:**

None Declared

